# Late Cervical Recurrence of Invasive Lobular Carcinoma Ten Years After Primary Breast Cancer: A Case Report and Review of the Literature

**DOI:** 10.3390/healthcare14020201

**Published:** 2026-01-13

**Authors:** Giulia Pellecchia, Stefano Restaino, Matteo Alfarè Lovo, Martina Arcieri, Monica Della Martina, Marco Petrillo, Giampiero Capobianco, Lorenza Driul, Giuseppe Vizzielli, The Gynecological Oncological Tumor Board Group

**Affiliations:** 1 Department of Medicine, University of Udine, 33100 Udine, Italy; giulia.pellecchia95@gmail.com (G.P.);; 2 Clinic of Obstetrics and Gynecology, “Santa Maria della Misericordia” University Hospital Azienda Sanitaria Universitaria Friuli Centrale, 33100 Udine, Italy; martina.arcieri@asufc.sanita.fvg.it (M.A.);; 3 PhD School in Biomedical Sciences, Gender Medicine, Child and Women Health, University of Sassari, 07100 Sassari, Italy; 4 Department of Medicine, Surgery and Pharmacy, University of Sassari, 07100 Sassari, Italy; 5 Institute of Pathology, “Santa Maria della Misericordia” University Hospital, Azienda Sanitaria Universitaria Friuli Centrale, 33100 Udine, Italy; 6 Department of Medical Oncology, “S. Maria della Misericordia” University Hospital, Azienda Sanitaria Universitaria Friuli Centrale (ASUFC), 33100 Udine, Italy; 7 Department of Oncological Radiotherapy, S.M. della Misericordia University Hospital, University of Udine, 33100 Udine, Italy; 8 Department of Obstetrics and Gynecology, Center for Fetal Care and High-Risk Pregnancy, University of Chieti, 66100 Chieti, Italy; 9 Department of Human Pathology in Adult and Childhood “G. Barresi”, University of Messina, 98125 Messina, Italy; 10 Department of Woman, Child and General and Specialized Surgery, University of Campania “Luigi Vanvitelli”, 80138 Naples, Italy; 11 Department of Medical and Surgical Sciences, Institute of Obstetrics and Gynaecology, University of Foggia, 71122 Foggia, Italy; 12 Department of Medicine, Surgery and Health Sciences, University of Trieste, 34149 Trieste, Italy

**Keywords:** breast cancer, recurrence, uterine cervical neoplasm, lobular carcinoma, case report, review literature

## Abstract

**Highlights:**

**What are the main findings?**

- **Cervical recurrence of invasive lobular breast carcinoma is exceptionally rare**, and this case underscores the importance of considering metastatic disease in patients presenting with gynecologic symptoms, even many years after initial breast cancer treatment.
- **The recurrence preserved the classic lobular immunohistochemical profile** (ER/PR positive, HER2 negative, E-cadherin negative, GATA3 and CK7 positive), reinforcing the diagnostic utility of IHC in distinguishing metastatic lobular carcinoma from primary cervical malignancies.
- **The patient experienced an unusually long disease-free interval** of nine years before cervical and multi-site recurrence, highlighting the unpredictable and frequently delayed metastatic patterns of lobular breast carcinoma.
- **PET-CT imaging showed a progressive and sustained treatment response**, supporting the effectiveness of CDK4/6 inhibition combined with aromatase inhibition (ribociclib + letrozole) in metastatic lobular breast cancer with gynecologic involvement.

**What are the implications of the main findings?**

- **This case emphasizes the importance of routine gynecologic assessment** and multidisciplinary surveillance in long-term follow-up of patients with lobular breast carcinoma, given their distinct metastatic behavior and potential for very late recurrence.

**Abstract:**

Invasive lobular carcinoma (ILC) accounts for approximately 15% of breast cancers and the most common neoplasm in the female population. Cervical involvement is exceptionally rare and often underrecognized. This relationship is well-defined in the context of breast and ovarian cancer syndrome related to BRCA gene mutations. However, it is also observed in rare but underreported cases of cervical metastases originating from breast cancer. The objective of this manuscript is to describe a rare case of cervical recurrence of invasive lobular carcinoma and summarize comparable case to guide future gynecologic follow-up strategies. Therefore, we report the case of a 60-year-old woman who developed a late cervical recurrence of ILC ten years after her initial breast cancer diagnosis. The patient had previously undergone mastectomy for ER-positive, PR-positive, HER2-negative ILC, followed by five years of adjuvant endocrine therapy. She remained disease-free until presenting with post-menopausal bleeding, urinary symptoms, and acute renal failure. Pelvic examination and ultrasonography revealed an enlarged, indurated cervix with bilateral hydroureteronephrosis. Biopsy demonstrated a discohesive infiltrate consistent with metastatic lobular carcinoma, confirmed by immunohistochemistry (GATA3+, CK7+, ER/PR+, E-cadherin−, CK20−, CDX2−). Staging PET-CT showed additional metastases involving bone, peritoneum, and lymph nodes. The patient began systemic therapy with ribociclib plus letrozole, achieving radiologic improvement of the cervical lesion and abdominal disease. After a follow-up of several months, she maintains stable disease but has persistent chronic renal impairment secondary to obstructive uropathy. This case highlights the ability of ILC to recur after long latency and to metastasize to unusual gynecologic sites such as the cervix. We also review the literature on cervical recurrence from lobular carcinoma to emphasize the importance of gynecologic surveillance in breast cancer survivors and to identify areas that require further investigation.

## 1. Introduction

Breast cancer is the most common malignancy in women, and invasive lobular carcinoma (ILC) represents approximately 15% of all cases [1]. Unlike invasive ductal carcinoma, ILC is characterized by its insidious growth pattern and a distinct metastatic profile, often involving unusual sites such as the peritoneum, gastrointestinal tract, and gynecologic organs. Metastatic involvement of the uterine cervix, however, is exceedingly rare, accounting for less than 1% of all gynecologic metastases, and is even more uncommon in the setting of ILC.

The most common site of breast cancer metastasis is the bone (75% cases of metastases), the lungs, the liver, and the brain [2].

Recurrence of extragenital cancers into the female genital tract is less common compared to genital tract cancers, with the ovaries being the most frequently involved site. Primary tumors usually originate from the breast and gastrointestinal tract due to the favorable lymphatic and nutrient environments these sites provide [3]. When an extragenital tumor metastasizes to the uterus, it is most found in the myometrium; however, in some cases, the metastasis is limited to the endometrium [3]. Even if they are relatively uncommon, up to 8% of breast carcinomas will metastasize to the uterus. Lobular carcinoma is the most frequent type of breast cancer that spreads to the uterus [4]. This may be due to the more common loss of the adhesive molecule E-cadherin in lobular carcinoma [5].

While metastasis to the uterus is rare, it is even more uncommon for breast cancer to spread to the cervix.

Because cervical metastasis may present with nonspecific gynecologic or urinary symptoms, diagnosis can be significantly delayed. Furthermore, when it occurs after a long disease-free interval, the possibility of metastatic recurrence may not initially be considered, leading to potential misclassification as primary cervical pathology. These diagnostic challenges highlight the importance of recognizing this metastatic pattern, especially in patients with a history of lobular breast cancer.

Montiel et al. conducted a systematic review of the literature in 2012, identifying 30 articles on breast cancer with cervical localization, corresponding to 36 patients and accounting for 4.5% of cases. Of these, half had a lobular histotype but astonishingly had the longest survival rates. The variability in reporting across published case series and isolated case reports limited the ability to accurately determine the clinical stage and initial treatment of the primary malignancies. Although it has been reported for several years, the literature still contains only highly heterogeneous case reports, leaving the susceptible patient’s etiopathogenetic mechanism and oncological profile unclear. Additionally, cervical metastasis sometimes appears at the onset of the disease and other times as a recurrence many years after the initial diagnosis, creating confusion and difficulty for clinicians in making a differential diagnosis. Therefore, we find it necessary to describe our case of cervical recurrence of lobular carcinoma of the breast ten years after the initial treatment and to summarize similar cases previously reported briefly [6].

Moreover, these factors, along with the observation that gastrointestinal and ovarian cancers most frequently lead to cervical metastases, support the hypothesis that a hematogenous pathway is responsible for this spread [7].

Although cervical involvement from breast cancer has been described for many years, the literature remains limited to highly heterogeneous case reports, leaving the underlying etiopathogenetic mechanisms and oncological characteristics poorly defined. Furthermore, cervical metastases may present either at initial diagnosis or as late recurrences, sometimes emerging many years after primary treatment, which complicates differential diagnosis for clinicians. For these reasons, we considered it essential to report our case of cervical recurrence of lobular breast carcinoma occurring ten years after the initial diagnosis and to provide a concise summary of similar cases previously published. The rationale for reporting this case lies in its rarity, its unusually long latency period, and the severe clinical consequences of cervical involvement, including obstructive uropathy and renal failure. Few cases describe such a presentation a decade after initial breast cancer treatment.

The aim of this report is to describe this rare late cervical recurrence of ILC, outline the diagnostic process including immunohistochemical differentiation, summarize treatment decisions, and contextualize the case within existing literature to improve clinical recognition.

## 2. Case Report

We present the case of a 60-year-old woman who was diagnosed with cervical metastasis secondary to lobular breast cancer after 10 years of primary diagnosis. Approval to publish the case was obtained from the patient and subsequently the study was approved by the local Institutional Review Board (RIF Prot IRB: 288/2024).

The patient had a history of chronic hypertension treated with lercanidipine and enalapril, anxiety managed with escitalopram, childhood allergic asthma treated with desensitization therapy, and a homozygous MTHFR mutation. She reported allergies to ramipril, amlodipine, and metoclopramide, and maintained an overall regular lifestyle. No relevant family history was noted. In 2014, the patient had been diagnosed with ER-positive (95% of cells), PR positive (95% of cells), Ki-67 positive (5–7% of cells), and HER-2 negative infiltrating lobular breast cancer. She underwent a total surgical mastectomy of the left breast and removal of the sentinel lymph node with final stage disease pT2 N1 (micrometastasis on sentinel lymph node) M0. The Tumour board discussion concluded with hormonal adjuvant therapy and counseling with the patient for short chemotherapy of 4 cycles with dynamic and cyclophosphamide (AC) or Taxotere and cyclophosphamide (TC). We discussed both options and their pros and cons with the patient, and she opted for hormonal therapy alone. She, therefore, took Tamoxifen for three years, then changed to Letrozole for another two years, ending in 2019. Oncological follow-up was then free of symptoms. Her upcoming history began with an urgent admission to the gynecology department due to acute renal failure and dependent edema, occurring in the context of atypical postmenopausal vaginal bleeding that had persisted for about six months and was under investigation. In her recent medical history, she also reported experiencing nocturia, frequent urination, and urinary incontinence for several months. At presentation to the Emergency Department, she reported hypogastric heaviness, urinary frequency, incontinence, and a pain NRS of 7/10. Examination revealed a globous abdomen with peripheral edema but no peritoneal signs; vital parameters were stable (BP 185/85 mmHg, HR 75 bpm, RR 14/min, SpO_2_ 98%, T 36 °C). Complete clinic and biochemical findings are reported in Supplementary Materials.

The gynecological examination revealed the following: the cervix was challenging to visualize, appeared epithelialized, increased in volume with a firm, fibrotic consistency, and had obliterated fornixes. There was an abundant hemorrhagic fluid discharge. Transvaginal ultrasonography showed an enlarged uterus (131 × 68 × 75 mm) with a 36 × 24 mm intramural-subserous leiomyoma, normal endometrial cavity, and no adnexal masses or pelvic effusion. An enlarged uterine cervix of approximately 4.5 cm, with an inhomogeneous echo structure, particularly at the posterior lip was also found. The color Doppler showed a CS3 color score, and the sliding sign was absent in both the anterior and posterior compartments.

**Figure 1:**
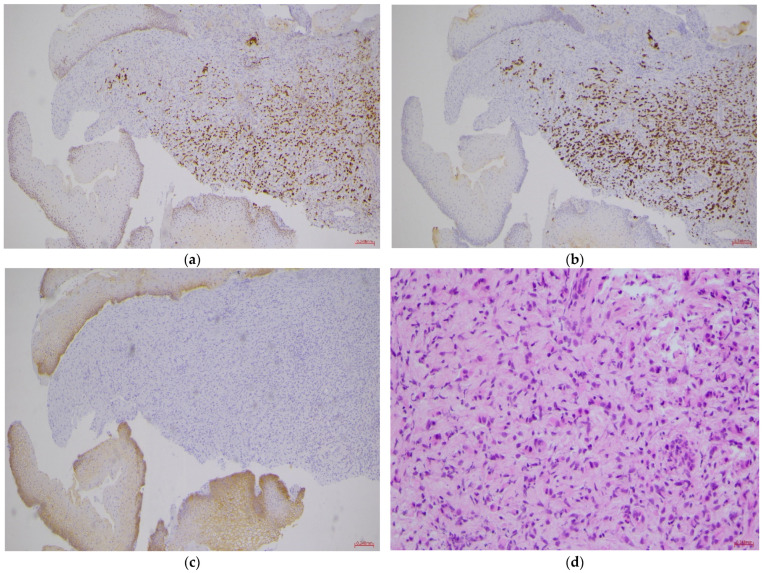
Histopathology and immunohistochemistry of the cervical lesion. Representative microphotographs showing features consistent with metastatic invasive lobular carcinoma. (**a**) GATA3-positive nuclei (IHC, ×200); (**b**) CK7-positive cytoplasmic staining (IHC, ×200); (**c**) Loss of E-cadherin expression (IHC, ×200); (**d**) Hematoxylin–eosin staining demonstrating classic single-file cell arrangement (H&E, ×250). Scale bars = 50 μm.

**Figure 2:**
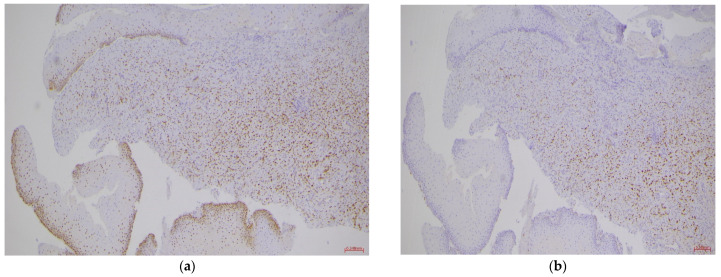
Hormone receptor profile of the cervical metastasis. (**a**) Strong ER nuclear positivity (IHC, ×200); (**b**) Strong PR nuclear positivity (IHC, ×200). Both findings are consistent with the patient’s original breast cancer phenotype.

Ultrasound also revealed bilateral hydroureteronephrosis, which was promptly managed in collaboration with nephrologists by placing bilateral pyelostomies. Given her oncologic history, differential diagnosis included new gynecologic disease versus metastatic recurrence. She underwent gynecologic examination under anesthesia with biopsies, which confirmed metastatic lobular breast carcinoma within 72 h. The pathologist reported fragments of mucosa lined with compound pavement epithelium devoid of atypia, with underlying chorion diffusely infiltrated by a proliferation of single epithelial cells, sometimes with cytoplasmic vacuoles, positive for GATA3 and Cytokeratin 7, and negative for Cytokeratin 20, CDX2, and E-Cadherin (Figure 1). Immunostaining results were as follows: Estrogen receptor: 90% positive cells; Progesterone receptor: 90% positive cells; Proliferation marker Ki-67: positive in 7% of tumor cells; HER2 status: negative with 20% of cells positive (Figure 2). The immunohistochemical procedure for hormone receptors was subject to external quality control UK NEQUAS—Breast Hormonal Receptor Module.

Staging PET-CT revealed further involvement of bone, peritoneum, and lymph nodes. A multidisciplinary discussion recommended first-line endocrine therapy with ribociclib and letrozole. Because of concurrent acute kidney injury, ribociclib was initiated at 400 mg/day (21 days on, 7 days off), with letrozole 2.5 mg/day. She received detailed counseling on toxicity monitoring, and laboratory tests were scheduled every two weeks, later monthly once stabilized. On hematochemical examination, CA15.3 was raised to 378.1 IU/mL; the last Pap test of a few months prior was completely negative.

PET-CT imaging showed widespread metastatic disease in March 2024. Subsequent scans demonstrated progressive metabolic response, with marked improvement by June 2024 and resolution of uterine and bone activity by October 2024. PET-CTs in March and June 2025 were negative at all previously involved sites. The most recent scan in October 2025 showed only nonspecific or likely inflammatory findings, with no clear evidence of active disease.

The most recent dosage of CA15.3 remains high at 178.4 UI/mL.

The renal damage caused by hydroureteronephrosis has led to grade IV chronic renal failure on obstructive genesis, and the patient is currently using pyelostomies. She is, therefore, still taking ribociclib at a reduced dosage.

Bilateral pyelostomies were maintained until renal function stabilized; they were removed but she continued nephrology follow-up for chronic renal impairment. Serial PET-CT scans showed progressive improvement: significant reduction of uterine and systemic disease, resolution of uterine and bony lesions, and complete metabolic remission of previously involved sites. After 3 months, ultrasound scan revealed only non-specific inflammatory changes. The patient experienced mild discomfort and temporary limitations while the pyelostomies were in place but returned to normal activities after their removal. She reported excellent treatment tolerance with no major side effects, and follow-up imaging and laboratory tests have shown no disease recurrence to date.

Regular oncologic follow-up continued every 14–30 days with laboratory and imaging surveillance every 3–4 months.

Regarding quality of life, the patient experienced temporary functional limitations due to the pyelostomies but returned to normal activities after their removal. She reported good tolerance to therapy and remained free of symptomatic recurrence. Psychologically, the diagnosis of recurrence caused initial distress and fear, for which she received and accepted prompt psychological support. Over time she became actively engaged in her therapeutic plan, expressing appreciation for the timely management and close monitoring.

At the last oncological check-up, the patient was clinically in discrete conditions: experiencing G1 asthenia and maintaining an appetite. She reported no nausea or vomiting, had regular digestion and had bowel movements regularized by laxatives. Diuresis was normal through pyelostomies with normal urine. She was apyretic with no suspicious respiratory symptoms. Occasional minimal vaginal bleeding persisted, but no bone pain was reported.

## 3. Discussion

This case described a rare recurrence of lobular carcinoma of the breast on the cervix.

The morphological pattern of lobular carcinoma—notably discohesive single cells and occasional intracellular vacuoles or signet-ring–like features—can closely mimic other adenocarcinomas and metastatic signet-ring tumors. Immunohistochemistry is therefore essential to distinguish metastatic lobular breast carcinoma from primary cervical neoplasms and from metastases of gastrointestinal or ovarian origin. Histologically the lesion showed discohesive single tumor cells with occasional cytoplasmic vacuoles, typical of lobular carcinoma. Immunostains were positive for GATA3 and CK7 and negative for CK20, CDX2 and E-cadherin, confirming metastatic lobular breast carcinoma rather than a primary cervical lesion. It is important to note that marker expression can be heterogeneous: mammaglobin and GCDFP-15 have variable sensitivity, and ER/PR may change under therapy; therefore, interpretation must integrate morphology, clinical history (including prior breast cancer), and radiologic staging. Although this clinical entity has been previously documented, it still needs to be better understood. Due to the heterogeneous nature of the case history, it is challenging to go beyond simple descriptions of individual cases. What is the profile of a patient with cervical metastasis or recurrence? From our analysis of the literature, it is evident that this condition is not limited to a single histotype or specific stage of the disease. The key to understanding it likely lies in studying its microenvironment.

It also appeared as IHC phenotypes from first diagnosis to cervical recurrence, remained the same even after ten years.

- Phenotype on primary (2014): ER 95%, PgR 95%, MIB-1 5–7%, HER-2 negative (IHC1+)

Phenotype on cervical recurrence (2024): ER 90%, PgR 90%, KI-67 7%, HER-2 1+ (20%). We find reason for this figure in the reported fact that half of recurrences of ER-positive tumors (like our patient’s cancer behavior) occur after at least 5 years [8].

Although both ILC and IDC are frequently HR-positive, ILC shows a distinct metastatic pattern with disproportionately more metastases to gynecologic organs. This is mainly attributed to loss of E-cadherin, which produces discohesive tumor cells capable of infiltrating stromal tissues such as the uterus and cervix. Reported cervical metastases from breast cancer are rare, but when they occur, ILC is far more common than IDC despite IDC being the dominant breast cancer subtype. Moreover, ILC is associated with late recurrences due to persistent ER/PR expression, consistent with the decade-long interval seen in our case. IDC, in contrast, metastasizes earlier and to more typical sites. These differences highlight a biological predilection of ILC for unusual metastatic sites such as the cervix.

Differently, diagnosis turned out to be different from the common case history: patient’s pap test had been completely negative at a prodromal stage of the onset of the abnormal uterine bleeding. This also contributed to the diagnostic delay in contrast to what has been reported in the literature with the finding of suspicious cytological atypia on the pap test which started further clinical exams [9,10].

This case reports a rare cervical recurrence of invasive lobular carcinoma (ILC) occurring ten years after initial breast cancer diagnosis. When compared with previously reported cases, the patient’s course demonstrates both concordant and distinctive features.

Consistent with the literature (Table 1), the tumor was hormone receptor-positive and maintained a stable immunophenotype over time. ER and PR expression remained high at recurrence, reflecting the known biology of ILC as a hormone-dependent disease with prolonged dormancy and a propensity for late relapse, often beyond five years. The latency observed in this case falls within the reported range of 2–15 years. Unlike many previously described patients with advanced primary disease, our patient was initially diagnosed at an early stage, confirming that cervical recurrence is not restricted to high-risk presentations.

Histologically, the cervical lesion showed typical lobular features with discohesive single cells and cytoplasmic vacuoles. As in other cases, immunohistochemistry was essential to distinguish metastatic ILC from primary cervical malignancies and other metastatic adenocarcinomas. Positivity for GATA3 and CK7 and loss of E-cadherin supported breast origin. In contrast to several reports in which abnormal Pap smears prompted diagnosis, cytology in our patient was negative, contributing to diagnostic delay and underscoring the limited sensitivity of cervical screening in this setting.

Cervical involvement occurred in the context of disseminated disease, consistent with literature indicating that cervical metastasis usually reflects systemic spread. The predilection of ILC for gynecologic sites is attributed to loss of E-cadherin, facilitating stromal infiltration and colonization of uncommon metastatic locations.

Therapeutically, this case differs from most historical reports by reflecting contemporary practice. Treatment with a CDK4/6 inhibitor combined with endocrine therapy was selected according to current guidelines and achieved effective disease control.

In summary, compared with previously reported cases, this patient illustrates the typical biological behavior of ILC—hormone receptor positivity, late recurrence, and gynecologic spread—while differing in initial stage and diagnostic pathway. This reinforces the need for long-term vigilance and prompt biopsy of cervical lesions in patients with a history of ILC, even many years after primary treatment.

Across reported cases, cervical recurrence from breast cancer occurred after long latency intervals (approximately 24–180 months), most commonly beyond five years from initial treatment. Invasive lobular carcinoma was the predominant histologic subtype, with consistent estrogen and progesterone receptor positivity at recurrence. Abnormal uterine bleeding was the most frequent presenting symptom, although incidental or asymptomatic cases were described. Cervical involvement was rarely isolated and usually associated with synchronous distant metastases, indicating systemic disease rather than a localized recurrence.

The diagnosis of cervical metastasis from invasive lobular breast carcinoma (ILC) relied on the integrated interpretation of imaging, histopathology, and immunohistochemistry (IHC). Although pelvic ultrasonography revealed only nonspecific uterine enlargement and leiomyoma, the patient’s clinical history prompted further evaluation. Biopsy was essential, as cervical metastases often mimic benign gynecologic conditions and are radiologically subtle. Histology showing discohesive cells with a linear “single-file” arrangement, together with IHC positivity for estrogen and progesterone receptors and E-cadherin loss, confirmed metastatic ILC rather than a primary cervical neoplasm. PET-CT contributed to staging by identifying extracervical disease, but its sensitivity for small-volume peritoneal and gynecologic involvement remains limited, representing a diagnostic constraint in such cases.

Therapeutic strategy was based on established evidence supporting CDK4/6 inhibitors combined with endocrine therapy as first-line treatment in HR-positive/HER2-negative metastatic breast cancer. Ribociclib plus letrozole was selected in accordance with national (AIOM) and international (NCCN) guidelines due to its demonstrated survival benefit, tolerability, and efficacy in controlling both visceral and bone disease. The patient’s renal impairment required initial dose adjustment, highlighting the importance of tailoring systemic therapy to organ function.

This case is notable for the exceptionally late recurrence—occurring ten years after the initial diagnosis—and the rare metastatic localization to the uterine cervix, a site involved in less than 1% of breast cancer metastases. Furthermore, the pattern of spread and peritoneal involvement is consistent with the known predilection of ILC for metastasizing to gynecologic and gastrointestinal sites, underscoring the need for clinicians to consider metastatic disease even in atypical presentations.

Limitations in the diagnostic process include the non-specificity of early imaging findings and the inherent difficulty of detecting lobular metastases radiologically, which may delay diagnosis. Additionally, this report is limited by its single-patient design, which restricts generalizability. Nonetheless, the case reinforces the value of maintaining a high index of suspicion in patients with prior ILC who present with gynecologic symptoms, even many years after primary treatment.

Clinically, this case highlights the importance of prompt biopsy of unusual cervical lesions in ILC survivors, the need for multidisciplinary evaluation, and the effectiveness of modern endocrine-targeted therapy in achieving durable metabolic remission. Future recommendations include routine consideration of metastatic disease in postmenopausal women with prior ILC and new cervical symptoms, and further research to clarify patterns of late metastatic spread and optimal surveillance strategies.

We conducted a review of the literature available on major search engines using the following keywords: lobular breast cancer*, tumor*, neoplasia*, carcinoma*, recurrence*, relapse*, uterine cervix*, metastasis*, cervix*. We identified 120 articles, nearly all of which were case reports, many quite dated, from 1980 to the present. We read the abstracts of all these articles and selected 25 of them, excluding those describing cases of metastasis at initial diagnosis and not at recurrency and/or cases of relapse or initial metastasis from histotypes other than lobular. From reading the full texts, 9 articles fully met our criteria: i.e., cases of recurrence of lobular carcinoma in the cervical site and not metastases at first diagnosis and/or from different histotypes. Case reports have also described secondary cervical localizations arising from infiltrating ductal carcinoma. Notably, the stages at initial breast cancer diagnosis were highly heterogeneous, preventing any meaningful conclusions about recurrence risk based on the characteristics of the primary tumor. Recurrence also occurs in stages I at diagnosis, as is our case. We proceeded to summarize included studied in Table 1 to facilitate a narrative analysis of the findings from similar cases already described.

Concerning immunohistochemistry, we also found the estrogen/progestin profile to be positive in all cases of recurrence and almost stable, where available, compared to that of breast diagnosis. The role these findings may have played in determining the site remains to be defined because, unfortunately, only single cases are available in the literature. Its periodic narrative review, however, makes it possible to compare them with each other and to identify foci for future research to understand their etiopathogenesis finally. On the other hand, its clinical manifestation itself lacks objective criteria: often, the onset symptom is abnormal uterine bleeding, and only sometimes has an early incidental diagnosis been reported through cytological abnormalities on the pap test.

In a series of 150 cases of extragenital cancers metastasizing to the female genital tract, the uterus was found to be the most resistant to metastasis, with only 8% of cases involving the uterus and merely 3.4% affecting the cervix [10]. Out of 52 cases metastasizing from breast cancer, none of them were localized to the cervix [10]. Potential explanations for this rarity include the cervix’s limited size, decreased blood flow and distal circulation, and the presence of abundant fibrous tissue, all of which make the uterine cervix may contribute to lower metastatic propensity [11].

Interestingly, these ILC are hormone-sensitive, and patients at younger ages are prone to gynecological metastases [11,12,13]. Furthermore, lower-grade and positive estrogen receptors are considered good prognostic factors [14].

An aura of uncertainty and the unknown surrounds this clinical entity, which appears to be more common than expected. Therefore, reporting individual cases and continuous review of the literature is essential in attempting to derive new insights into its prevention and treatment.

For all these reasons, a summary of cases from the literature of cervical recurrence of breast lobular carcinoma was performed, and it is provided in Table 1. The aim was to highlight clinical and histopathological features of similar cases, narrow the gap of the unknown, and reduce the heterogeneity that keeps this oncological entity in the shadows. As anticipated, these are all case reports.

It results as all the patients were young and had received hormone therapy with tamoxifen at the time of their initial diagnosis. Except for two studies [10,12] where data are unavailable, immunohistochemistry was positive for estrogen and progesterone receptors in all tumor phenotypes.

The latency period from initial treatment to relapse ranges between 2 and 15 years. The initial stage of the disease is advanced or includes at least lymph node micrometastases, except for two studies [7,15] who had isolated cervical recurrence and, from there, extended to the uterus and left ovary, respectively. There is no consistent data regarding onset symptoms, as each case is presented differently. The most common symptom, however, was abnormal uterine bleeding.

The “seed and soil” hypothesis, proposed by Stephen Paget, is widely accepted although it is just a theoretical, as a model of metastasis for breast cancer. This model suggests that successful colonization of a secondary organ requires both the intrinsic properties of the tumor cells (the “seed”) and a compatible, supportive microenvironment (the “soil”) [16].

In this molecular interaction, the secondary localization of the cervix also is involved, despite its seemingly hostile environment; however, this remains an area requiring further investigations. Scientific evidence is currently limited to individual case reports and review of the remainders so far available, given the high heterogeneity because of the rarity of the event that limits study designs. For sure, a preclinical in vitro study examining the microscopic basis of this seed and soil interaction in determining lobular breast cancer’s cervical metastasis would be beneficial.

One of the most recent papers on the subject, from 2019, summarizes the literature by counting approximately 35 cases reported up to that time. However this number also includes cases with an infiltrating ductal histotype; overall timing of occurrence ranged from 24 to 180 months [2].

Yagizi et al. reviewed first 24 patients with this rare condition [17]. Over two-thirds experienced vaginal bleeding as a presenting symptom, and more than 60% showed no signs of disease during the examination. Therefore, the metastasis would have been overlooked if comprehensive evaluations, including Pap smear, colposcopy, and biopsy, had not been conducted.

In most cases, cervical metastasis of breast cancer indicates widespread disease. In 67–89% of reported cases, distant metastases at other sites were present when cervical metastasis was diagnosed [18]. The cervix’s response to metastatic disease involves fibrous proliferation and an inflammatory cellular reaction, which may explain the clinical finding of an enlarged, hardened cervix which is often described among papers [2]. From their literature review, albeit it was just published in 2017, it appears that the case histories involved are of rather old work and mostly of cases of metastatic cervical disease at onset. In our case, it is instead a recurrence 5 years after the end of primary hormonal treatment and with a lower initial stage than those reported.

In addition, the finding of cervical disease right during antihormonal treatment with tamoxifen is reported. This represents another difference from our case in that the patient had completed the therapy about five years before [19]. However, its own value in the context of the disease is still unknown.

Ustaalioglu et al. described a case of recurrence of ILC on cervix during anastrozole therapy, which has no adverse effects on endometrium as observed with tamoxifen [20]. It is therefore plausible to assume, but remains to be proven, that there is no direct correlation with the use of antihormonal therapy in the determination of this disease location.

Moving to diagnostic work up, surely immunohistochemistry (IHC) panel is essential for distinguishing between primary and metastatic cervical malignancies [21]: CK7+/CK20-pattern in fact is typical of breast cancers [22].

Instead, Scott et al. presents a case series of four instances of squamous cell carcinoma of the cervix, detailing the cytological and histological features of acantholysis and its resemblance to the lobular malignant histotype of breast cancer, characterized by dedifferentiation as an aggressive behavior of a cervical primary tumor. Three out of four cases had no positive history of lobular carcinoma of the breast. In any case, it testifies to how much this is still a field that needs to be investigated, reported, and studied to understand its mechanisms [23].

Several authors concluded that metastasis to the uterine cervix should be considered in women with a history of breast cancer, especially when they exhibit abnormal vaginal bleeding; and the gynecological examination should be included in the regular oncological follow up of these patients [2,24].

For the American Society of Clinical Oncology (ASCO), all patients with breast carcinoma should be under regular gynecologic surveillance. Summarizing the insights gained from individual cases and their comparison with selected cases, we suggest the need for a gynecological follow-up for these patients, not only during antihormonal therapy with tamoxifen—as some guidelines propose, though not consistently or uniformly—but also for an extended period after the completion of treatment. A gynecological examination first, combined with routine Pap smear screening, albeit with its limitations already discussed, could facilitate the early detection of this unusual disease presence, even in the absence of clear symptoms. Currently, with regard to these latter, there is no definitive guidance on this approach. Finally, the impact of omitting the short cycle of chemotherapy on disease recurrence cannot be determined conclusively. Even in similar cases documented in the literature, where patients were diagnosed at stage I and received adjuvant chemotherapy, cervical recurrence still happened [25].

**Table 1:** Case reports of cervical recurrence of lobular breast cancer.

Study	Age at R	Stage at Diagnosis of Lobular Breast Cancer (FIGO Stage and/or Surgical TNM)	IHC at Diagnosis	First Treatment	Time to Cervical Recurrence	Symptoms and Signs (Markers, etc.)	Type of Recurrence (Isolated, Multiple); Sites	IHC of Cervical Recurrence	Recurrence Treatment
Lim et al., 2021 [26]	57 y	IIIC	ER (+) PR (+), Her2 Neu (−)	Left mastectomy + SLN; CHT + RT + tamoxifen	24 mo	Post menopausal AUB + right breast mass	Multiple sites metastases	CK7(+), ER(+), GATA3(+); CD10(−); CK20(−); CDX2(−)	RH+BSO Fulvestrant; ribociclib
Silva Fontinele et al., 2019 [17]	57 y	T4bN1M0; IIIb	ER(+); PR(+); HER2(−); Ki67:20%; E-cadherin (−)	NACT; left radical mastectomy+ axillary emptying; adjuvant RT + tamoxifen	39 mo	AUB; pap smear: LSIL; colposcopy(−)	Multiple sites metastases (bones)	Cytokeratin(+); cadherin(−); ER(+); PR(+); HER2(−)	RH+BSO; anastrozole.
Seo et al., 2017 [15]	46 y	T1N0M0	ER(+); PR(+); C-ErbB2/ Kras:(+);Ecadherin (−)	NACT+ Left breast conservative surgery+ axillary lymph nodes; adjuvant CHT+ RT + Tamoxifen and goserelin	24 mo	AUB with anemia; incidental finding of cervical leiomyoma; CA125: 535.4	Multiple. (uterus and left ovary)	ER(+); PR(+); cytokeratin (+);	RH+BSO; CHT
Razia et al., 2017 [12]	58 y	IIIc (IDC with large groups of ILC)	NA	Surgery + CHT+ RT + hormone therapy (goserelin acetate, tamoxifen, toremifene)	108 mo	AUB; endometrial thickness and myometrial myoma	Multiple (cervix and myoma)	ER(+); PR(+); HER2 (+); E-cadherin (−).	RH+BSO+ partial colectomy for IOP involvement
Lokadasan et al., 2015 [27]	49 y *	T3N2M0	Weak ER(+); PR(+)	Radical mastectomy + CHT+RT+ tamoxifen		Abdominal distension and pain; pedal and abdominal edema; ascites; omental cake; cervix mass; bilateral ovarian masses; bulky aortic nodes; stomach thickening	Multiple (bilateral ovaries, omental deposits)	Cytokeratin (+); E-cadherin (−); ER(+); PR(+)	CHT
Waks et al., 2015 [28]	53 y	T2N1M0	ER (+) PR (+)	Breast conservative surgery + axillary lymph nodes dissection; CHT+RT; tamoxifen	180 mo	AUB	Multiple (uterus, para-aortic lymph nodes)	P63 (−); p16 (−); Her2 neu: (−) ER (+) weak; PR (+)	CHT
Perisic et al., 2007 [29]	65 y	T2N1M0	ER(+); PR(+);	Radical mastectomy + axillary lymph nodes; CHT + RT+ tamoxifen	52 mo	Incidental finding of suspicious change in cervix	Isolated	GCDFP-15(+); CK7:(+); CEA (+)	RH+BSO CHT planned (patient refused; she died of PD)
Manci et al., 2008 [7]	41 y	T1N0M0	ER(+); PR(+); C-erb2: (−); Ki67 (+)	Quadrantectomy+ axillary nodes dissection; RT + tamoxifen	132 mo	Progressive uptake of CA15.3 up to 161 value. Asymptomatic Pap test: (−). Incidental polypoid endocervical mass	Isolated	ER (+): PR (+); C-erb2 (−); Ki67: (+).	RH + systematic pelvic lymphadenectomy
Rau et al., 2003 [10]	55 y	IIIB	NA	Radical mastectomy; CHT + RT; adjuvant tamoxifen	48 mo	Pain abdomen; pyometra; indurated cervix infiltrating bladder and vaginal vault; enlarged ovaries; Pap smear: (+) for tumor cells	Isolated	NA	Patient refused
This paper	60 y	T2N1M0	ER (+): PR (+); MIB: 7%; HER2 (−).	Radical mastectomy + bilateral SLN + Hormone therapy (tamoxifen and letrozole)	108 mo	AUB, discharge, cervical mass; hydronephrosis with renal failure; edema uptake CA15.3	Multiple (bones, peritoneal)	ER (+); PR (+); Ki67: 7%; HER2: 1+ (20%).	Ribociclib

**Legenda: Review of the literature of all cases of cervical recurrences from breast cancer**. Age at R = age at recurrence; IHC = immunohistochemistry; SLN = sentinel lymph node; HER2/neu = human epidermal growth factor receptor 2; (+) = positive; (−) = negative; CHT = chemotherapy; RT = radiotherapy; mo = months; AUB = abnormal uterine bleeding; ER = estrogen receptor; PR = progesterone receptor; RH = radical hysterectomy; BSO = bilateral salpingo-oophorectomy; NACT = neoadjuvant chemotherapy; NA = not available; GCDFP-15 = gross cystic disease fluid protein-15; IDC = invasive ductal carcinoma; ILC = invasive lobular carcinoma; IOP = intraoperative finding; PD = progressive disease. * Lokadasan et al. (2015) [27] included two cases; Case 1 was excluded due to uncertain recurrence status.

## 4. Conclusions

This case illustrates that invasive lobular carcinoma can recur many years after initial treatment and may metastasize to uncommon sites such as the uterine cervix. Persistent or unexplained gynecologic symptoms in breast cancer survivors should prompt consideration of metastatic disease, even when imaging findings are subtle. Early biopsy, integration of histopathology with IHC, and multidisciplinary assessment remain essential for timely diagnosis. The favorable response to ribociclib plus letrozole also underscores the effectiveness of contemporary endocrine-targeted therapy in managing late metastatic recurrences. Clinicians should maintain vigilance during long-term follow-up of ILC patients and remain aware of atypical metastatic patterns to optimize early detection and care.

The description of these isolated cases is essential to assist clinicians in finding a management comparison, as they cannot yet rely on high-quality, standardized evidence. The key will be to study the mechanisms of the primary tumor microenvironment both in vitro and within the cervix to understand how it can localize in such an apparently irrational site. A crucial aspect that emerged, warranting further study, is the potential involvement of tamoxifen in the mechanism of metastasis determination, as it was the common factor in all reported cases.

## Data Availability

The data presented in this study are available on request from the corresponding author. All data presented in the study are derived from the clinical records of a single patient and cannot be made publicly available due to privacy and ethical considerations.

## Supplementary Materials

The following supporting information can be downloaded at: https://www.mdpi.com/article/10.3390/healthcare14020201/s1, Figure S1: Timeline of the disease’ course (initial diagnosis, treatment, recurrence, management, out-comes); Figure S2: Biochemical outcome assessment: Table S1: Hematological exams; Table S2: Tumor markers trend across the timeline; Table S3: Immunohistochemistry findings at recurrence.
