# Supplementary material for: Late Cervical Recurrence of Invasive Lobular Carcinoma Ten Years After Primary Breast Cancer: A Case Report and Review of the Literature
Source: Healthcare (Basel). 2026 Jan 13;14(2):201. doi: 10.3390/healthcare14020201 (PMC12840778; doi:10.3390/healthcare14020201)
Supplement: Supplementary file 1 [file healthcare-14-00201-s001.zip › healthcare-3983949-supplementary.pdf]

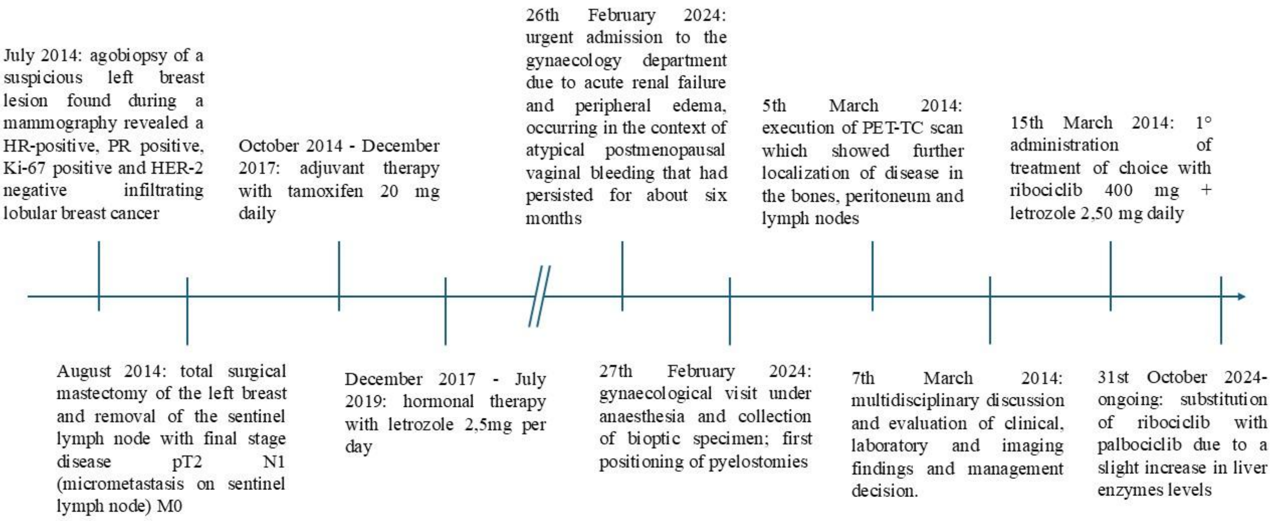

Figure S1. Timeline of the disease' course (initial diagnosis, treatment, recurrence, management, outcomes).

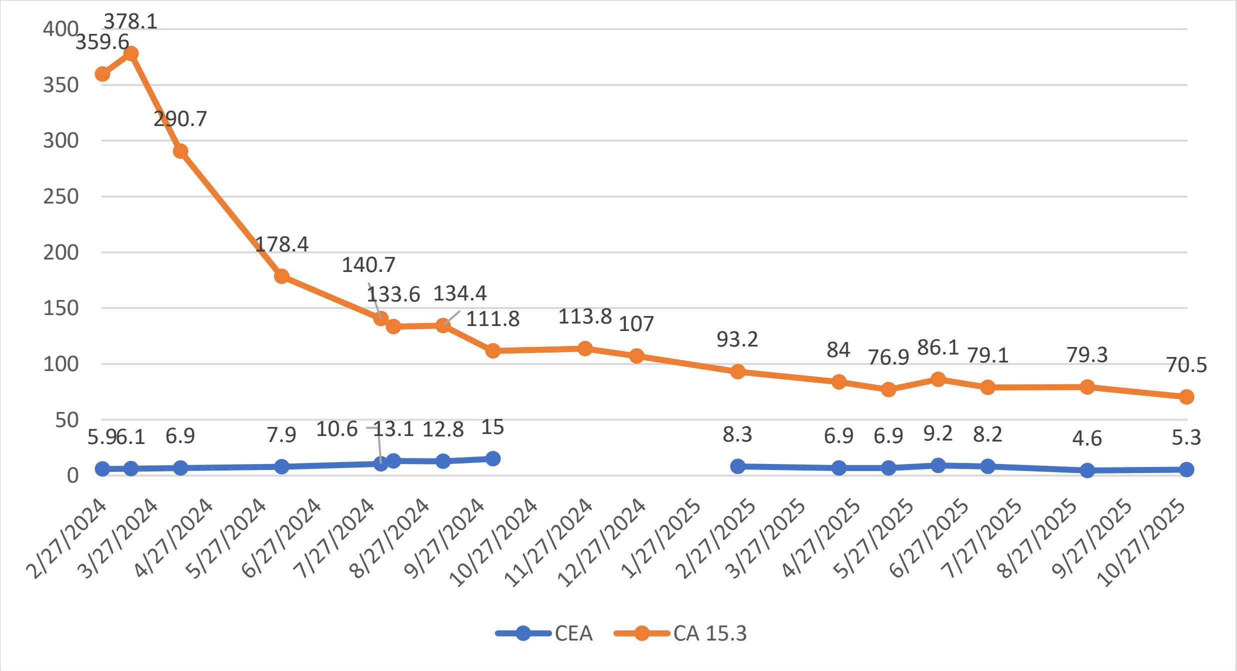

Figure S2. Biochemical outcome assessment.

Table S1. Haematological exams.

	Value	Metric	Reference range from our Laboratory Department
**White Blood Cells**	**5,98**	**x10^3/µL**	**4,00 – 11,00**
**Red Blood Cells**	**2,63**	**x10^6/µL**	**4,20 – 5,00**
**Hemoglobin**	**8,4**	**g/dL**	**12,0 – 16,0**
**Hematocrits**	**24,2**	**%**	**37,0 – 50,0**
**Platelet count**	**211**	**x10^3/µL**	**150 – 400**
**Urea nitrogen**	**64**	**mg/dL**	**6 – 20**
**Creatinine**	**4,74**	**mg/dL**	**0,51 – 0,95**
**eGFR (CKD EPI)**	**9**	**mL/min/1,73mq**	**CKD classification<br>Stage 1+2: GFR > 60<br>Stage 3: GFR 30 – 59<br>Stage 4+5: GFR < 30**
**Sodium**	**140**	**mMol/L**	**135 – 145**
**Potassium**	**5,43**	**mMol/L**	**3,50 – 5,10**
**Chlorides**	**108**	**mMol/L**	**96 – 109**
**Calcium**	**2,29**	**mMol/L**	**2,15 – 2,50**
**Gamma-glutamyl transferase**	**9**	**UI/L**	**6 – 39**
**Aspartate aminotransferase**	**13**	**UI/L**	**4 – 32**
**Alanine aminotransferase**	**12**	**UI/L**	**4 – 33**
**Bilirubin (total)**	**0,21**	**Mg/dL**	**0,20 – 1,00**
**Bilirubin (direct)**	**0,10**	**Mg/dL**	**0,00 – 0,30**
**Serous albumin**	**39**	**g/L**	**35 – 50**
**CEA**	**5,9**	**ng/mL**	**Physiological threshold: < 3<br>Pathological threshold: > 10**
**CA 19.9**	**10,8**	**UI/mL**	**Physiological threshold: < 31<br>Pathological threshold: > 60**
**CA 15.3**	**359,6**	**U/mL**	**1,0 – 35,0**
**CA 125**	**38,9**	**U/mL**	**1,5 – 35,0**

Table S2. Tumor markers trend across the timeline.

	CEA	CA 15.3
27/02/2024	5,9	359,6
14/03/2024	6,1	378,1
11/04/2024	6,9	290,7
07/06/2024	7,9	178,4
02/08/2024	10,6	140,7
09/08/2024	13,1	133,6
06/09/2024	12,8	134,4
04/10/2024	15	111,8
25/11/2024	Not calculated	113,8

24/12/2024	Not calculated	107
19/02/2025	8,3	93,2
17/04/2025	6,9	84
15/05/2025	6,9	76,9
12/06/2025	9,2	86,1
10/07/2025	8,2	79,1
04/09/2025	4,6	79,3
30/10/2025	5,3	70,5

Table S3. Immunohistochemistry findings at recurrence.

Marker	Result
Estrogen receptor	90% positive cells
Progesterone receptor	90% positive cells
Proliferation marker Ki-67	positive in 7% of tumor cells
HER2 status	negative with 20% of cells positivity
